# Expression of Serum sLOX-1 in Patients with Non-Small-Cell Lung Cancer and Its Correlation with Lipid Metabolism

**DOI:** 10.1155/2022/6619331

**Published:** 2022-04-11

**Authors:** Fangfang Hao, Jinliang Chen, Jinnan Wu, Xin Ge, Xuedong Lv, Dongmei Zhang, Jianrong Chen

**Affiliations:** ^1^Department of Respiratory Medicine, Affiliated Hospital 2 of Bengbu Medical College, Bengbu, China; ^2^Department of Respiratory Medicine, Affiliated Hospital 2 of Nantong University, Nantong, China; ^3^Department of Respiratory Medicine, Affiliated Hospital of Nantong University, Nantong, China; ^4^Medical Research Center, Affiliated Hospital 2 of Nantong University, Nantong, China

## Abstract

**Objective:**

The aim of this study was to investigate the expression level of soluble LOX-1 (sLOX-1) in the serum of non-small-cell lung cancer (NSCLC) patients and its correlation with lipid metabolism.

**Methods:**

99 inpatients with NSCLC and 81 healthy controls were enrolled in this study. The levels of serum sLOX-1 were compared between the two groups, and the correlation of sLOX-1 with clinicopathological characteristics, blood lipid indices, and carcinoembryonic antigen was analyzed.

**Results:**

Compared with the healthy controls, sLOX-1, low-density lipoprotein, triglyceride, and carcinoembryonic antigen in the patients with NSCLC were significantly higher (*p* < 0.05), while the expression level of high-density lipoprotein was lower (*p* < 0.05). The expression level of sLOX-1 in the serum of patients with healthy controls was positively correlated with low-density lipoprotein (*r* = 0.72, *p* < 0.05). The levels of sLOX-1 and low-density lipoprotein in the serum of patients with NSCLC were closely related to the lymph node metastasis, distant metastasis, and TNM stage (*p* < 0.05). Compared with a single index, when the sLOX-1 was combined with the CEA, its specificity increased significantly to 97.5% (AUC = 0.995, *p* < 0.01, 95% CI: 0.989–1.000).

**Conclusion:**

sLOX-1 and low-density lipoprotein were overexpressed in the serum of patients with NSCLC, positively correlated, and closely related to the TNM stage and metastasis. This result suggested that lipid metabolic disorders may promote the progression of NSCLC through sLOX-1, which could be a potential serological marker with diagnostic value for NSCLC.

## 1. Introduction

The incidence of lung cancer, the most common cancer, is increasing. Lung cancer is a major public health problem worldwide and is one of the main causes of cancer-related deaths in the world [1, 2]. According to pathological classification, lung cancer can be divided into nonsmall (NSCLC) and small-cell lung cancers, and NSCLC accounts for approximately 86% [3]. The 5-year survival rate of patients with lung cancer is only about 15%, which is largely because no specific clinical symptoms are observed in the early stages of lung cancer. As such, most patients are in the middle and late stages at the time of treatment [2, 3]. Low-dose computed tomography (LDCT), a test method with a lower radiation dose than conventional CT, is currently the main means of early screening for lung cancer, but can lead to a high false positive rate, excessive follow-up examinations, invasive procedures, and patients' own anxiety [4]. Therefore, finding sensitive and easily obtained tumor markers that reflect the occurrence and development of tumors is of great significance for the early diagnosis of NSCLC.

The metabolic reprogramming is a hallmark of cancer [5]. One of the common phenotypes that tumors possess is that cells can divide and proliferate rapidly and uncontrollably. Thus, tumors need a large supply of energy and materials to meet their own growth and proliferation [5]. In addition to relying on glucose and amino acid metabolic pathways, highly proliferative cancer cells exhibit strong lipid and cholesterol affinities, which are met by increasing the uptake of exogenous lipids and lipoproteins or overactivating their endogenous synthesis (i.e., lipogenesis and cholesterol synthesis) [5]. Besides, studies show that the dysregulation of lipid metabolism plays a crucial role in the process of membrane synthesis, energy production, and signal transduction of cancer cells and participates in regulating various carcinogenic processes, including tumor cell growth, migration, and metastasis formation [5, 6]. Many lipid-related metabolic genes, including OLR-1 that encodes lectin-like oxidized low-density lipoprotein receptor-1 (LOX-1), are overexpressed in different cancer cell lines [7].

LOX-1 belongs to the C-type lectin-like receptor family [7]. In 1997, LOX-1 was first discovered in aortic endothelial cells and was initially considered to be the main receptor for oxidized low-density lipoprotein (oxLDL) in endothelial cells [8]. The receptor is also expressed in monocytes, macrophages, smooth muscle cells, platelets, and kidney, lung, and neuronal tissues [9–11]. LOX-1 is a type II transmembrane glycoprotein with a molecular weight of 50 kD and belongs to the C-type lectin family structurally and to the E-type scavenger receptor family functionally [12]. The LOX-1 consists of four domains: the N-terminal cytoplasmic, the transmembrane, the associated neck, and the extracellular C-terminal lectin-like domains (CTLD) [11, 12]. Among these domains, the CTLD with a highly conserved structure is the key region for LOX-1 to recognize multiple ligands and mediate internalization and phagocytosis, and the N-terminal glycosylation can regulate protein folding in the endoplasmic reticulum, secretory transport to the plasma membrane, and ligand recognition [13]. The human LOX-1 has two forms, namely, membrane-bound and soluble LOX-1. The membrane-bound LOX-1 is highly expressed in the bone marrow, spinal cord, lung, and placenta and moderately expressed in the skeletal muscle, heart, artery, and ovary. The soluble LOX-1 exists in blood, and its plasma concentration is related to the expression level of the membrane-bound receptor [14]. LOX-1 can be cleaved to form soluble LOX-1 (sLOX-1) in the proximal extracellular region of the cell membrane, and the expression level of LOX-1 can be reflected by the concentration of sLOX-1 in circulation [15]. LOX-1 is highly expressed in prostate, colorectal, gastric, pancreatic, and breast cancers and other tumor tissues or cells, which are closely related to tumor staging, invasion, and metastasis [16–20]. In 2015, Jiang *L* et al. reported that the LOX-1 immunohistochemical staining score combined with body mass index can predict the poor prognosis of patients with NSCLC and squamous cell carcinoma [21]. However, the level and the clinical significance of sLOX-1 in the serum of patients with NSCLC have not been reported. Therefore, this study aims to investigate the expression level of serum sLOX-1 in patients with NSCLC and its correlation with lipid metabolism and explore the value of sLOX-1 in the diagnosis of NSCLC.

## 2. Subjects and Methods

### 2.1. Subjects

A total of 590 inpatients who were visiting as outpatients had suspicion of lung cancer from the Department of Thoracic and Cardiac Surgery, Respiratory and Critical Care Medicine of the Affiliated Hospital 2 of Nantong University from September 2018 to December 2020. According to the inclusion and exclusion criteria set in this study, 99 patients with NSCLC were finally enrolled (43 males and 56 females; average age 62.87 ± 10.93 years). The TNM stage of each carcinoma sample was determined in accordance with the criteria of the WHO and the UICC TNM staging system (8th edition) [22]. All patients met the following eligible criteria: (1) all patients had a pathologically confirmed diagnosis of NSCLC with no previous or coexisting cancer; (2) all enrolled patients did not receive any surgical treatment or take drugs affecting lipid levels before venous blood collection, such as taking hormones (methylprednisolone), lipid-lowering drugs (atorvastatin), hypoglycemic drugs (metformin and acarbose), antihypertensive drugs (diuretics, *β*-blockers, and calcium ion antagonists), antiepileptic drugs (phenytoin sodium), or other drugs known to affect lipid metabolism; (3) patients with concomitant diseases that were associated with increasing serum lipid levels (i.e., hyperlipidemia, coronary atherosclerotic heart disease, cerebrovascular accident, liver disease) were excluded; and (4) patients taking hormone replacement therapy or any drug known to affect lipid metabolism were excluded. At the same time, 81 healthy volunteers who underwent physical examination (36 males and 45 females; an average age of 60.00 (38.50–67.50) years) were included in the control group. The inclusion criteria for the control group were as follows: (1) negative LDCT examination; (2) no clinical symptoms of lung cancer (such as cough, hemoptysis, dyspnea, and weight loss); (3) no family history of malignant tumors; (4) no major organ disease, such as heart, liver, spleen, lung, and kidney; (5) no history of hyperlipidemia, coronary heart disease, cerebral infarction, hypertension, diabetes mellitus, or other diseases; and (6) patients who did not take hormones or any other drug known to affect lipid metabolism. All samples were collected with the approval of the Medical Ethics Committee of the Second Affiliated Hospital of Nantong University and with the informed consent of patients.

### 2.2. Measurement of the Lipid Metabolism Indices and the Carcinoembryonic Antigen (CEA) Level

Fasting venous blood was collected from the subjects on the first day when they were admitted to the hospital for various examinations, diagnosis, and treatment. The levels of triglyceride (TG), total cholesterol (TC), high-density lipoprotein (HDL), low-density lipoprotein (LDL), and CEA in serum were detected using an automatic biochemical analyzer. The normal ranges of each index were as follows: TG, 0.2–2.0 mmol/L; TC, 2.9–6.0 mmol/L; HDL, 1.1–1.7 mmol/L; LDL, 1.55–3.35 mmol/L; and CEA, 0–10 ng/mL.

### 2.3. Determination of sLOX-1 in Serum

On the first day of admission, 5 mL of venous blood was drawn from patients with NSCLC, placed in the coagulation tube, and centrifuged at 3500 rpm for 5 minutes. The supernatant from the coagulation tube was collected, placed in the RNase-free centrifuge tube, and stored at −80°C for further testing. The samples from healthy people were the same as those from patients with lung cancer. The serum sLOX-1 concentration was determined using the human LOX-1 ELISA kit, which adopts the sandwich ELISA principle. The serum was analyzed in accordance with the manufacturer's instructions (Abcam, ab212161).

### 2.4. Statistical Analysis

All statistical analyses were performed using IBM SPSS Statistics 25.0, and mapping was performed using the GraphPad Prism 8.0 software. All the data were tested for normal distribution. The data with normal distribution were expressed as the mean ± standard deviation (‾*χ* ± *s*), whereas the data with nonnormal distribution were expressed as median (quartile range; *M* [QU-QL]). The *T*-test was used to compare two groups of samples, and the analysis of variance was used to compare multiple groups of samples. The Pearson test was used for correlation analysis. The sLOX-1 diagnostic value and the CEA expression levels in serum for lung cancer were analyzed using the ROC curve, and the area under the curve (AUC) was compared using the *Z* test. *p* < 0.05 suggested a significant difference.

## 3. Results

### 3.1. Baseline Characteristics of Patients with NSCLC and Normal Controls

This study included 99 hospitalized patients with NSCLC, including 43 males and 56 females. 36 patients had a history of smoking. Among the patients with NSCLC, 80 adenocarcinomas and 19 squamous carcinomas were observed. In accordance with the TNM stage, 49 patients had stages I–II and 50 patients with stages III–IV NSCLC, respectively, and 43 and 26 patients had lymph node and distant metastases, respectively. In addition, 81 healthy volunteers who underwent physical examinations were included in the same period. There is no significant difference in age, sex, and smoking history was observed between patients with NSCLC and normal controls (*p* > 0.05; Table 1).

### 3.2. Expression of sLOX-1, Lipid Metabolism, and CEA in Serum of Two Groups

Compared with the healthy controls, the expression levels of serum sLOX-1, LDL, TG, and CEA in patients with NSCLC were significantly higher (*p* < 0.05; Table 2), whereas the expression level of HDL in patients with NSCLC was lower than that in normal controls (*p* < 0.05; Table 2).

**Figure 1:**
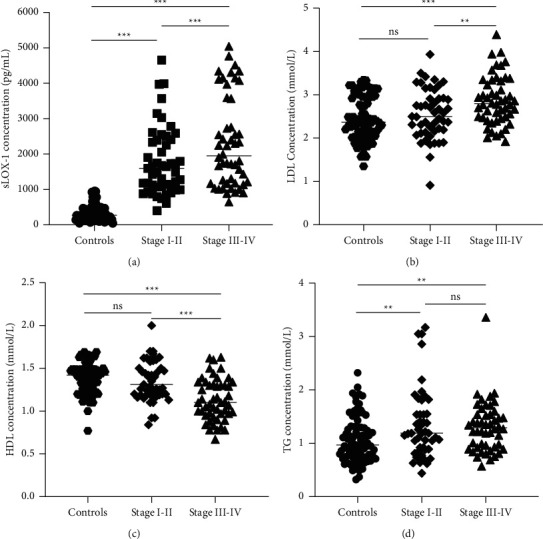
Expression levels of sLOX-1, LDL, HDL, and TG in serum of patients with NSCLC stage I–II and stage III–IV. ns: no significance.^*∗∗*^*p* < 0.01. ^*∗∗∗*^*p* < 0.001.

The expression level of sLOX-1 in the serum of patients with stages I–II and III–IV NSCLC was significantly higher than that of healthy controls (*p* < 0.001), and the expression level of sLOX-1 in the stage III–IV NSCLC group were higher than the stage I–II NSCLC group (*p* < 0.001; Figure 1(a)). In accordance with the trend of sLOX-1, the LDL concentration in the serum of patients with stage III–IV NSCLC was significantly higher than that of healthy controls and patients with stage I–II NSCLC group (*p* < 0.01; Figure 1(b)). In addition, the concentration of HDL in the serum of patients with stage III–IV NSCLC was significantly higher than that of healthy controls and patients with stage I–II NSCLC (*p* < 0.01), whereas there was a significant difference in serum HDL expression between stage I–II and stage III–IV (*p* < 0.05; Figure 1(c)). From Figure 1(d), it can be found that the concentration of TG in serum of patients with stage I–II and stage III–IV was significantly higher than that of healthy controls (*p* < 0.05).

### 3.3. Correlation among sLOX-1, Lipid Indexes, and CEA

**Figure 2:**
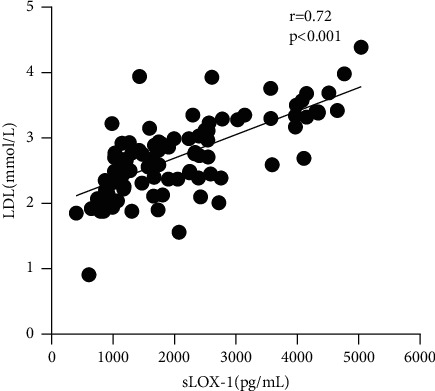
Correlation of serum sLOX-1 with blood lipid LDL.

The expression level of sLOX-1 in the serum of NSCLC was positively correlated with LDL (*r* = 0.72, *p* < 0.001; Table 3 and Figure 2) but not with HDL, TG, TC, and CEA (*p* > 0.05; Table 3).

### 3.4. Correlation among sLOX-1, LDL, and Clinicopathological Features

In this study, the serum sLOX-1 and LDL levels in patients with NSCLC were closely related to lymph node metastasis, distant metastasis, and TNM stage (*p* < 0.001; Table 4) but not to sex, age, smoking history, and histological type (*p* > 0.05; Table 4).

### 3.5. Diagnostic Efficacies of sLOX-1, CEA, and sLOX-1 Combined with CEA for NSCLC

**Figure 3:**
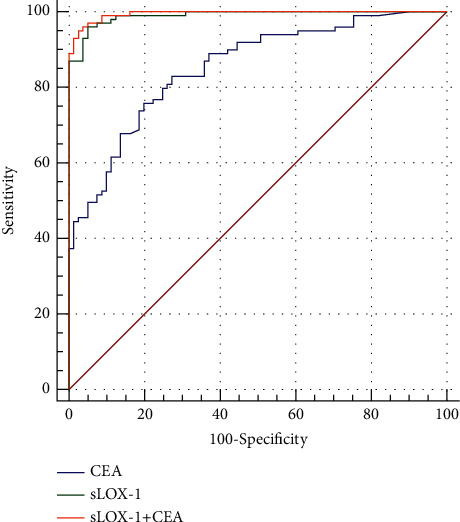
The diagnostic value of sLOX-1 and CEA single index and combination for NSCLC.

ROC curves were used to analyze the diagnostic efficacies of sLOX-1, CEA, and their combined indices. It can be found from Table 5 and Figure 3 that for a single index, the diagnostic value of serum sLOX-1 (AUC: 0.990 and sensitivity and specificity of 96.0% and 95.1%, respectively) was significantly higher than that of CEA (sensitivity and specificity of 82.8% and 72.8%, respectively, and AUC: 0.854). When sLOX-1 was combined with CEA, which was higher than the diagnostic value of single sLOX-1 and CEA, and its specificity was 97.5% (AUC = 0.995, *p* < 0.05, 95% CI: 0.989–1.00).

## 4. Discussion

With the rapid growth and aging of the global population, cancer has become increasingly prominent as the leading cause of death and the most common cancer [3]. In recent years, the metabolic reprogramming has become a hallmark of cancer. Among them, the dysregulation of lipid metabolism plays an important role in the occurrence and the development of tumors [5, 6]. This study aims to investigate the expression level of the lipid receptor sLOX-1 in the serum of patients with NSCLC, its correlation with blood lipid metabolism, and its diagnostic value in NSCLC.

Previous studies have shown that LOX-1 is highly expressed in gastric and prostate cancers and other tumor tissues, thereby promoting the proliferation, migration, and invasion of tumor cells and enhancing tumor angiogenesis and lymph node metastasis [23–25]. In 2020, Japanese scholars reported for the first time that the serum level of sLOX-1 in patients with colorectal cancer is higher than that in control patients, which is a marker of poor prognosis [26]. But reports on the relationship between LOX-1 and lung cancer are lacking. Jiang *L* et al. reported for the first time in 2015 that the LOX-1 immunohistochemical staining score in tumor tissues combined with BMI can predict poor prognosis in patients with NSCLC and squamous cell carcinoma [21]. In 2019, Wang et al. have reported that LOX-1 expression is promoted by the TGF-*β*-C/EBP*δ*-Slug pathway, which enhances the ability of lung adenocarcinoma cells to uptake oxLDL [27]. Our study is the first to find that the expression level of LOX-1 in the serum of patients with NSCLC is significantly increased and associated with the distant metastasis of tumors, lymph node metastasis, and TNM stage. This indicates that sLOX-1 is helpful to judge the progression of NSCLC. Compared with the healthy controls, the level of sLOX-1 in stage I–II NSCLC group was significantly increased (*p* < 0.001), which means that the high expression of sLOX-1 has certain auxiliary value for the diagnosis of NSCLC. Pucci et al. [20] found that LOX-1 and its splice variant LOX-1Δ4 were highly expressed in breast cancer, and their expression patterns could be specifically regulated in different breast cancer phenotypes while affecting the proliferation rate and apoptosis of breast cancer cells, death induction, DNA repair process, and epigenetic status of cancer cells. Zeya et al. [11] found that LOX-1 interacts with its ligands to induce the expression of inflammatory cytokines such as IL-1*β* and TNF-*α*. In addition, studies have found that IL-1*β* promotes the invasion and metastasis of lung cancer A549 cell line, thereby promoting the progression and metastasis of cancer, and TNF-*α* could promote the metastasis of lung cancer by inducing EMT [28, 29]. Studies have shown that LOX-1 participates in the cross presentation of antigens, exerts the targeted effect of immune activation and antigen transmission, inhibits the attack of the immune system, and promotes tumor progression [11]. Murdocca et al. [17] found that the downregulation of LOX-1 expression in colorectal cancer cell lines strongly affected the presence of the volatile compound butyrate, which resulted in a significant increase in histone H4 acetylation. The change of acetylation pattern suggests that LOX-1 may be involved in epigenetic regulation of tumor suppressor gene transcription. This also explains that the high expression of LOX-1 is closely related to the progression of NSCLC. Further analysis has revealed that the expression level of serum sLOX-1 in patients with NSCLC is closely related to the lipid metabolism and significantly positively correlated with the serum LDL level.

Our study has found that the expression levels of sLOX-1 and LDL in the serum of patients with NSCLC are significantly higher than those in healthy controls, whereas the HDL expression level in the serum of patients with NSCLC is significantly lower than that of normal controls. The serum LOX-1 expression level is closely related to the LDL expression level. LDL and HDL, which are used to reflect the level of lipid metabolism, are the most widely used laboratory indicators in clinical practice. Increasing evidence shows that disorders of lipid metabolism can promote the growth and proliferation of gastric, breast, and prostate cancers and other tumor cells, which is related to their occurrence, development, treatment, and prognosis evaluation [30–32]. Our study confirms a lipid metabolic disorder in patients with NSCLC. Compared with the healthy controls, patients with NSCLC has significantly higher serum LDL level (*p* = 0.035) and significantly lower HDL level (*p* = 0.003), which are abnormally correlated with the TNM stage. LDL is the key lipoprotein carrier from cholesterol to cancer cells [33], and sLOX-1 is a lipoprotein receptor. Both of them play a certain role in promoting the abnormal lipid metabolism of tumor cells, which can also indirectly explain the conclusion that sLOX-1 is positively correlated with the level of LDL in this study. The level of oxidative stress in patients with tumor is significantly elevated, and LDL is easily oxidized to form oxLDL [7]. Studies have shown that oxLDL can stimulate survival pathways by promoting the epithelial-mesenchymal transition (EMT), inducing protective autophagic responses, activating inflammasomes, and releasing growth factors, cytokines, and other proinflammatory markers. Moreover, oxLDL can induce mutations, stimulate tumor cell proliferation, trigger metastasis, and induce treatment resistance [34, 35].

Studies have found that the oxLDL/LOX-1 activates the NF-*κ*B signaling pathway through a variety of pathways, regulates the transcription of a variety of genes, and induces the increased expression of VEGF, MMP-2, and MMP-9, thereby promoting tumor growth, migration, and invasion through effective blood vessels [7, 23, 25, 35, 36]. Also, LOX-1 can increase the intracellular ROS level, cause oxidative DNA damage, and induce apoptosis by binding to oxLDL bodies [7, 34, 35]. In addition, oxLDL/LOX-1 promotes the invasion and the migration of cancer cells by inducing actin skeleton remodeling and migration by promoting EMT [25]. Many studies have shown that LOX-1 promotes the proliferation, migration, and invasion of a variety of tumor cells and promotes tumor angiogenesis and lymph node metastasis [16–19]. By binding to its ligand, LOX-1 can activate NOX/MAPKs/NF-*κ*B or PI3K/Akt/GSK3*β* signaling pathways, regulate the expression of genes such as inflammation, hypoxia, and oxidative stress, and then promote tumor growth, migration, and invasion by effective new blood vessels [7–9]. In addition, LOX-1 also participates in the cross presentation of antigens, exerts the targeted effect of immune activation and antigen transmission, inhibits the attack of the immune system, and promotes the occurrence and progression of tumors [37–39]. Therefore, we infer that LOX-1 can activate the cell signaling pathway in the body to promote the malignant progression of NSCLC by binding to its ligand. Our results suggest that the disorder of lipid metabolism in the body may promote the occurrence and development of NSCLC through sLOX-1, but the mechanism that plays a role remains to be further explored.

In addition, our study showed that TG was highly expressed in the serum of patients with NSCLC, which was consistent with the findings of Ulmer et al. [40]. Previous studies have found that high levels of TG increase the overall risk of cancer by 20% [41]. Studies have found that inflammatory factors such as TNF-*α* and IL-1 can promote the synthesis of triglycerides in hepatocytes, and inflammation can reduce the clearance of TG-rich particles [42]. Different metabolites produced in the process of the triglyceride/free fatty acid cycle can act as signaling molecules, affect the activities of various transcription factors, enzymes, or receptors, help regulate cell survival, proliferation, movement, etc., and then play a certain role in tumor progression [43]. However, the specific mechanism is still unclear and needs further research and exploration.

The CEA, an acidic glycoprotein with the characteristic determinant of human embryonic antigen, belongs to the tumor-associated antigen and is also the earliest lung cancer-related tumor marker [44]. Our study has found that the diagnostic value of the serum sLOX-1 is significantly higher than that of CEA in terms of a single index. Moreover, the specificity of sLOX-1 is significantly increased when combined with CEA, indicating that serum sLOX-1 can be used as a new potential biomarker for the diagnosis of NSCLC.

## 5. Conclusion

In conclusion, sLOX-1 and LDL are significantly highly expressed in the serum of patients with NSCLC and are closely related to the lymph node metastasis, distant metastasis, and TNM stage of NSCLC. The expression level of sLOX-1 is positively correlated with LDL, which indicates that lipid metabolic disorders may promote the progression of NSCLC by affecting LDL through sLOX-1. sLOX-1 is expected to be a potential serological marker with diagnostic value for NSCLC.

## Figures and Tables

**Table 1:** Baseline characteristics of total patients, NSCLC patients, and healthy controls.

Variables	NSCLC patients	Healthy controls	*P* value
*Gender*			
Male	43	36	0.891
Female	56	45	

*Age(years)*			
<63	49	46	0.329
≥63	50	35	

*Smoking history*			
Yes	36	29	0.937
No	63	52	

Histology			
Squamous cell carcinoma	19	–	–
Adenocarcinoma	80	–	

*Lymph Node Metastasis*			
Yes	43	–	–
No	56	–	

*Distant Metastasis*			
Yes	26	–	–
No	73	–	

*TNM stage*			
I–II	49	–	–
III–IV	50	–	

**Table 2:** The expression levels of sLOX-1, blood lipid metabolism indexes, and CEA in the serum of two groups of patients.

Variables	Normal range	NSCLC patients	Healthy controls	*P* value
sLOX-1(pg/mL)	-	2066.00 (1146.00–2565.00)	330.20 (162.50–451.30)	<0.001
LDL (mmol/L)	1.55–3.35	2.72 ± 0.59	2.47 ± 0.51	<0.01
HDL (mmol/L)	1.1–1.7	1.27 (1.05–1.42)	1.38 (1.21–1.49)	<0.001
TG (mmol/L)	0.2–2.0	1.37 (0.86–1.54)	1.12 (0.75–1.32)	<0.05
TC (mmol/L)	2.9–6.0	4.31(3.83–4.76)	4.19 (3.83–5.76)	0.72
CEA (ng/mL)	0–10	3.33 (2.01–13.19)	1.10 (0.65–1.85)	<0.01

**Table 3:** Correlation of serum sLOX-1 with blood lipid indexes and CEA.

Variables	sLOX-1	
	*r*	*p*
LDL	0.72	<0.001
HDL	0.15	0.15
TG	-0.10	0.35
TC	-0.02	0.85
CEA	0.08	0.42

**Table 4:** Correlation between sLOX-1, LDL, and clinicopathological characteristics of NSCLC patients.

Variables	sLOX-1 (pg/mL)		*p* value	LDL (mmol/L)		*p* value
	<1733.0	≥1733.0		<2.715	≥2.715	
Gender						
Male	20	23	>0.38	19	24	0.15
Female	31	25		33	23	

Age (years)						
<63	26	23	0.76	26	23	0.92
≥63	25	25		26	24	

BMI						
<23.3	31	25	0.38	33	23	0.15
≥23.3	20	23		19	24	

Smoking history						
Yes	10	15	>0.18	9	16	0.06
No	41	33		43	31	

Histology						
Squamous cell carcinoma	9	10	0.69	8	12	0.21
Adenocarcinoma	42	38		44	35	

Lymph node metastasis						
Yes	12	31	<0.01	16	27	<0.01
No	39	17		36	20	

*Distant Metastasis*						
Yes	12	14	<0.01	9	17	<0.05
No	39	34		43	30	

*TNM stage*						
I–II	35	14	<0.01	32	17	<0.01
III–IV	16	34		20	30	

**Table 5:** The diagnostic value of sLOX-1 and CEA single index and combination for NSCLC.

Variables	AUC	*p* value	95% CI	Sensitivity (%)	Specificity (%)
sLOX-1	0.990	*p* < 0.01	0.981–0.999	96.0	95.1
CEA	0.854	*p* < 0.05	0.800–0.907	82.8	72.8
sLOX-1+CEA	0.995	*p* < 0.01	0.989–1.000	94.9	97.5

## Data Availability

No data were used to support this study.

## Abbreviations

NSCLC: Non-small-cell lung cancer
LDCT: Low-dose computed tomography
LOX-1: Lectin-like oxidized low-density lipoprotein receptor-1
oxLDL: Oxidized low-density lipoprotein
sLOX-1: Soluble LOX-1
CEA: Carcinoembryonic antigen
TG: Triglyceride
TC: Total cholesterol
HDL: High-density lipoprotein
LDL: Low-density lipoprotein
CTLD: C-terminal lectin-like domains
EMT: Epithelial-mesenchymal transition.

## References

[1] Siegel R. L., Miller K. D., Jemal A. (2019). Cancer statistics, 2019. *CA: A Cancer Journal for Clinicians*.

[2] Barta J. A., Powell C. A., Wisnivesky J. P. (2019). Global epidemiology of lung cancer[J]. *Ann Glob Health*.

[3] Nasim F., Sabath B. F., Eapen G. A. (2019). Lung cancer. *Medical Clinics of North America*.

[4] Wang X., Liu H., Shen Y., Li W., Chen Y., Wang H. (2018). Low-dose computed tomography (LDCT) versus other cancer screenings in early diagnosis of lung cancer. *Medicine*.

[5] Beloribi-Djefaflia S., Vasseur S., Guillaumond F. (2016). Lipid metabolic reprogramming in cancer cells. *Oncogenesis*.

[6] Maan M., Peters J. M., Dutta M., Patterson A. D. (2018). Lipid metabolism and lipophagy in cancer. *Biochemical and Biophysical Research Communications*.

[7] Balzan S., Lubrano V. (2018). LOX-1 receptor: a potential link in atherosclerosis and cancer. *Life Sciences*.

[8] Kume N., Murase T., Moriwaki H. (1998). Inducible expression of lectin-like oxidized LDL receptor-1 in vascular endothelial cells. *Circulation Research*.

[9] Jin P., Cong S. (2019). LOX-1 and atherosclerotic-related diseases. *Clinica Chimica Acta*.

[10] Singh S., Gautam A. S. (2019). Upregulated LOX-1 receptor: key player of the pathogenesis of atherosclerosis. *Current Atherosclerosis Reports*.

[11] Zeya B., Arjuman A., Chandra N. C. (2016). Lectin-like oxidized low-density lipoprotein (LDL) receptor (LOX-1): a chameleon receptor for oxidized LDL. *Biochemistry*.

[12] Navarra T., Del Turco S., Berti S., Basta G. (2010). The lectin-like oxidized low-density lipoprotein receptor-1 and its soluble form: cardiovascular implications. *Journal of Atherosclerosis and Thrombosis*.

[13] De Siqueira J., Abdul Zani I., Russell D. A., Wheatcroft S. B., Ponnambalam S., Homer-Vanniasinkam S. (2015). Clinical and preclinical use of LOX-1-specific antibodies in diagnostics and therapeutics. *Journal of Cardiovascular Translational Research*.

[14] Chen X.-P., Du G.-H. (2007). Lectin-like oxidized low-density lipoprotein receptor-1: protein, ligands, expression and pathophysiological significance. *Chinese Medical Journal*.

[15] Murase T., Kume N., Kataoka H. (2000). Identification of soluble forms of lectin-like oxidized LDL receptor-1. *Arteriosclerosis, Thrombosis, and Vascular Biology*.

[16] Wan F., Qin X., Zhang G. (2015). Oxidized low-density lipoprotein is associated with advanced-stage prostate cancer. *Tumor Biology*.

[17] Murdocca M., Mango R., Pucci S. (2016). The lectin-like oxidized LDL receptor-1: a new potential molecular target in colorectal cancer. *Oncotarget*.

[18] Li C., Zhang J., Wu H. (2017). Lectin-like oxidized low-density lipoprotein receptor-1 facilitates metastasis of gastric cancer through driving epithelial-mesenchymal transition and PI3K/Akt/GSK3*β* activation. *Scientific Reports*.

[19] Zhang J., Zhang L., Li C. (2018). LOX-1 is a poor prognostic indicator and induces epithelial-mesenchymal transition and metastasis in pancreatic cancer patients. *Cellular Oncology*.

[20] Pucci S., Polidoro C., Greggi C. (2019). Pro-oncogenic action of LOX-1 and its splice variant LOX-1Δ4 in breast cancer phenotypes. *Cell Death & Disease*.

[21] Jiang L., Jiang S., Lin Y. (2015). Combination of body mass index and oxidized low density lipoprotein receptor 1 in prognosis prediction of patients with squamous non-small cell lung cancer. *Oncotarget*.

[22] Lim W., Ridge C. A., Nicholson A. G., Mirsadraee S. (2018). The 8th lung cancer TNM classification and clinical staging system: review of the changes and clinical implications. *Quantitative Imaging in Medicine and Surgery*.

[23] Ma C., Xie J., Luo C. (2019). Ox-LDL promotes lymphangiogenesis and lymphatic metastasis in gastric cancer by upregulating VEGF-C expression and secretion. *International Journal of Oncology*.

[24] González-Chavarría I., Fernandez E., Gutierrez N. (2018). LOX-1 activation by oxLDL triggers an epithelial mesenchymal transition and promotes tumorigenic potential in prostate cancer cells. *Cancer Letters*.

[25] González-Chavarría I., Cerro R. P., Parra N. P. (2014). Lectin-like oxidized LDL receptor-1 is an enhancer of tumor angiogenesis in human prostate cancer cells. *PLoS One*.

[26] Nakashima-Nakasuga C., Hazama S., Suzuki N. (2020). Serum LOX-1 is a novel prognostic biomarker of colorectal cancer. *International Journal of Clinical Oncology*.

[27] Wang D., Cheng X., Li Y. (2020). C/EBP*δ*-Slug-Lox1 axis promotes metastasis of lung adenocarcinoma via oxLDL uptake. *Oncogene*.

[28] Voronov E., Apte R. N. (2017). Targeting the tumor microenvironment by intervention in interleukin-1 biology. *Current Pharmaceutical Design*.

[29] Liu W., Chen X., He Y. (2019). TNF-*α* inhibits xenograft tumor formation by A549 lung cancer cells in nude mice via the HIF-1*α*/VASP signaling pathway[J]. *Oncology Reports*.

[30] Sun H., Huang X., Wang Z. (2019). Triglyceride-to-high density lipoprotein cholesterol ratio predicts clinical outcomes in patients with gastric cancer. *Journal of Cancer*.

[31] Martin L. J., Melnichouk O., Huszti E. (2015). Serum lipids, lipoproteins, and risk of breast cancer: a nested case-control study using multiple time points. *JNCI Journal of the National Cancer Institute*.

[32] Wang F. M., Zhang Y. (2019). High lipoprotein level is independently associated with adverse clinicopathological features in patients with prostate cancer[J]. *Disease Markers*.

[33] Cruz P. M. R., Mo H., McConathy W. J., Sabnis N., Lacko A. G. (2013). The role of cholesterol metabolism and cholesterol transport in carcinogenesis: a review of scientific findings, relevant to future cancer therapeutics. *Frontiers in Pharmacology*.

[34] Zhou T., Zhan J., Fang W. (2017). Serum low-density lipoprotein and low-density lipoprotein expression level at diagnosis are favorable prognostic factors in patients with small-cell lung cancer (SCLC). *BMC Cancer*.

[35] Bitorina A. V., Oligschlaeger Y., Shiri-Sverdlov R., Theys J. (2019). Low profile high value target: the role of OxLDL in cancer. *Biochimica et Biophysica Acta (BBA) - Molecular and Cell Biology of Lipids*.

[36] Dandapat A., Hu C., Sun L., Mehta J. L. (2007). Small concentrations of oxLDL induce capillary tube formation from endothelial cells via LOX-1-dependent redox-sensitive pathway. *Arteriosclerosis, Thrombosis, and Vascular Biology*.

[37] Liu B., Li S., Xiu B. (2019). C-terminus of heat shock protein 60 can activate macrophages by lectin-like oxidized low-density lipoprotein receptor 1. *Biochemical and Biophysical Research Communications*.

[38] Delneste Y., Magistrelli G., Gauchat J.-F. (2002). Involvement of LOX-1 in dendritic cell-mediated antigen cross-presentation. *Immunity*.

[39] Zurawski G., Zurawski S., Flamar A.-L. (2016). Targeting HIV-1 env gp140 to LOX-1 elicits immune responses in rhesus macaques. *PLoS One*.

[40] Ulmer H., Borena W., Borena W. (2009). Serum triglyceride concentrations and cancer risk in a large cohort study in Austria. *British Journal of Cancer*.

[41] Melvin J. C., Holmberg L., Rohrmann S., Loda M., Van Hemelrijck M. (2013). Serum lipid profiles and cancer risk in the context of obesity: four meta-analyses[J]. *J Cancer Epidemiol*.

[42] Esteve E., Ricart W., Fernández-Real J. M. (2005). Dyslipidemia and inflammation: an evolutionary conserved mechanism. *Clinical Nutrition*.

[43] Gong Y., Dou L. J., Liang J. (2014). Link between obesity and cancer: role of triglyceride/free fatty acid cycling. *European Review for Medical and Pharmacological Sciences*.

[44] Grunnet M., Sorensen J. B. (2012). Carcinoembryonic antigen (CEA) as tumor marker in lung cancer. *Lung Cancer*.

